# Serum‐derived exosomes from mice with highly metastatic breast cancer transfer increased metastatic capacity to a poorly metastatic tumor

**DOI:** 10.1002/cam4.575

**Published:** 2016-01-04

**Authors:** Reginald M. Gorczynski, Nuray Erin, Fang Zhu

**Affiliations:** ^1^ Toronto General HospitalUniversity Health NetworkTorontoOntarioCanada; ^2^ Faculty of MedicineDepartment of ImmunologyInstitute of Medical ScienceUniversity of TorontoTorontoOntarioCanada; ^3^ Department of Medical PharmacologySchool of MedicineAkdeniz UniversityAntalyaTurkey

**Keywords:** Breast cancer, CD200:CD200R, immunotherapy, metastasis, serum factors

## Abstract

Altered interaction between CD200 and CD200R represents an example of “checkpoint blockade” disrupting an effective, tumor‐directed, host response in murine breast cancer cells. In CD200R1KO mice, long‐term cure of EMT6 breast cancer, including metastatic spread to lung and liver, was achieved in BALB/c mice. The reverse was observed with 4THM tumors, an aggressive, inflammatory breast cancer, with increased tumor metastasis in CD200R1KO. We explored possible explanations for this difference. We measured the frequency of circulating tumor cells (CTCs) in peripheral blood of tumor bearers, as well as lung/liver and draining lymph nodes. In some cases mice received infusions of exosomes from nontumor controls, or tumor bearers, with/without additional infusions of anticytokine antibodies. The measured frequency of circulating tumor cells (CTCs) in peripheral blood was equivalent in the two models in WT and CD200R1KO mice. Increased metastasis in EMT6 tumor bearers was seen in vivo following adoptive transfer of serum, or serum‐derived exosomes, from 4THM tumor bearers, an effect which was attenuated by anti‐IL‐6, and anti‐IL‐17, but not anti‐TNF *α*, antibody. Anti‐IL‐6 also attenuated enhanced migration of EMT6 cells in vitro induced by 4THM serum or exosomes, or recombinant IL‐6. Exosome cytokine proteomic profiles responses in 4THM and EMT6 tumor‐bearing mice were regulated by CD200:CD200R interactions, with attenuation of both IL‐6 and IL‐17 in 4THM CD200^tg^ mice, and enhanced levels in 4THM CD200R1KO mice. We suggest these cytokines act on the microenvironment at sites within the host, and/or directly on tumor cells themselves, to increase metastatic potential.

## Introduction

Breast cancer is recognized to be the most prevalent malignancy for women, with significant impact on both quantity and quality of life. Conventional therapies, predominantly surgery, radiation, and/or chemotherapy, have played a major role in helping to control disease over the past decades, without leading to long‐term cure. As in other malignancies, immunotherapy offers the hope of a different and novel approach, but oncologists and immunologists alike have long appreciated that its success likely depends on a better understanding of the mechanisms(s) governing self‐tolerance, which likely will have to be overcome for any efficacy to be achieved with this approach. Current research has focused on improved understanding of the nature and effective presentation of tumor antigens, as well as appreciating the need to define the host elements which can contribute to an effective immune response, and the factors which in turn may regulate this. Current thought is that immunologic “cure” of cancer depends on development of an effective T‐cell‐mediated immune response, following antigen presentation to naïve T cells by dendritic cells (DCs) 1. It should be noted, however, that mAbs also represent a highly successful class of immunologic therapeutics used in cancer, including those directed at HER‐2 (e.g., in breast cancer), CD20 (e.g., in CLL), and VEGF in many solid tumors 2. Their mechanism(s) of action include disruption of cell signaling cascades, direct or indirect tumor killing, and more recently their incorporation as chimeric antigen receptors (CARs) into autologous (naïve) T cells for immunotherapy as reported recently in hematologic malignancies 3.

Failure of activation of T‐cell immunity may reflect a problem with antigen presentation, with the available naïve T‐cell receptor repertoire (a consequence of immune editing) 4, limited costimulatory signaling 5, T‐cell suppression within the tumor microenvironment 6, and the action of ligand:receptor pairs which regulate immune reactivity at key “checkpoints”^7^, ^8^, ^9^. We have explored in some detail the role for, and importance of, one such ligand:receptor pair, CD200:CD200R1, in the regulation of growth and metastasis in murine breast cancer, using EMT6 10 and 4THM (originally derived from heart metastasis of 4T1 cells 11, 12) tumor models. The EMT6 breast cancer line forms tumors in BALB/c female mice with limited metastatic potential, and moderate immunogenicity, while in contrast, the 4THM tumor is a poorly immunogenic and very aggressive tumor, metastasizing to brain, liver, lungs, and marrow. In the EMT6 model, increased immunosuppression and attenuation of an inflammatory response (by increased signaling through CD200:CD200R1 in CD200^tg^ mice, or with CD200^tg^ tumor cells 13) led to increased metastasis, while the reverse was the case in CD200R1KO mice 14. Somewhat surprisingly, these same manipulations produced the opposite effect in 4THM tumor‐bearing mice, with decreased growth in CD200^tg^ and increased growth/metastasis in CD200R1KO 15, consistent with the hypothesis that for this tumor increased inflammation promoted growth/metastasis of 4THM, unlike EMT6.

Epithelial‐to‐mesenchymal transition (EMT), a hallmark of early cancer growth/metastasis, is regulated by an inflammatory cytokine milieu 16, 17, 18, 19. Within the tumor microenvironment, many cells, including tumor‐associated macrophages (TAMs), dendritic cells, and tumor infiltrating lymphocytes, are potential sources of these inflammatory cytokines, and many of these are reported to contribute to growth/expansion of cancer stem cells, CSCs, the subpopulation within a tumor which likely represents the tumor initiating/maintaining population 19. High levels of the inflammatory cytokine IL‐6 are correlated with poor prognosis in breast cancer 17, and IL‐6 trans‐signaling has been suggested to affect directly EMT6 and 4T1 breast cancer cell aggressiveness, leading to increased metastasis 20.

We have explored the role of altered local (tumor) and systemic (plasma) inflammatory cytokine levels in wild‐type (WT) and CD200R1KO mice on the levels of circulating tumor cells, CTCs, as well as on microscopic metastases in draining lymph nodes (DLNs), as predictive markers for measured macroscopic metastasis in EMT6 and 4THM. In addition, we asked whether plasma‐derived exosomes were a source of inflammatory cytokines potentially responsible for altered metastatic growth in these models.

## Materials and Methods

### Ethics review

All experimental studies described herein were approved by a local institutional review board, certified by the Canadian Council on Animal Care (protocol AUP#1.15).

### EMT6 and 4THM tumor models and analysis of micro‐/macrometastasis

Both EMT6 and 4THM tumors were grown following injection of 2 × 10^5^ cells into the mammary fat pad of BALB/c female mice (WT or CD200RKO). Except where specified, tumors were resected at 14 days and 10 days later macrometastases to lung/liver were enumerated visually on tissues harvested and fixed in Bouin's solution. Micrometastases to draining lymph nodes (DLNs) were also measured at this time on cells harvested from individual mice and cloned at limiting dilution in microtitre plates for 21 days as described previously 13. In this case, a mAb (clone 35C1) for a breast‐cancer‐amplified kinase (BTAK‐1) obtained from Hycult Biotechnology (5405 PB Uden, the Netherlands) was used with permeabilized cloned cells to confirm tumor cells in all colonies as discussed in earlier reports 13, 14.

Where the frequency of circulating tumor cells (CTCs) was assessed in peripheral blood (PBL), PBL were harvested by cardiac puncture and spun over mouse hypaque (Cedarlane Labs, Hornby, Ontario) to deplete red cells. CD45^−^ cells (obtained as the eluate after passage twice over a CD45^+^ enrichment column; Miltenyi, San Diego, CA) were used as a surrogate population containing CTCs, and were cloned at limiting dilution on irradiated (3000 Rads) feeder layers of BALB/c bone marrow (5 × 10^5^/well), with colonies enumerated at 21 days. Again tumor cells in PBL‐derived colonies were confirmed by staining with anti‐BTAK‐1 13.

### Serum‐derived exosomes for injection into mice and cytokine profile analysis

Exosomes were isolated from serum samples using a commercial kit (Invitrogen: Cat#4478360) as per the manufacturer's instructions. When serum/exosomes were pooled from 4THM/EMT6 tumor‐bearing mice, a minimum of 20 donors were used at 16 days post tumor injection. Subsequent total exosome protein isolation was performed on isolated exosomes using a separate kit from the same manufacturer (#4478545), again following instructions as provided. Quantitation of exosome preparation, and standardization between different exosome samples, used a commercial cytokine ELISA kit (CD63 ExoELISA, catalog# EXOEL‐CD63A‐1) from System Biosciences (distributed by MJS Biolynx Inc., Brockville, ON, Canada). Exosomes for injection into mice were resuspended in cold PBS (4°C) to the same volume as the serum from which they were derived. Where serum or exosomes were injected into mice animals received 150 *μ*L of material at 48 h intervals beginning on the day of tumor inoculation, for a total of six injections. When mice in addition received normal rabbit serum (NRS), or anti‐IL‐6, anti‐IL‐17, or anti‐TNF*α* (all purchased from MyBiosource.ca), animals were injected with 50 *μ*g of antibodies at 60 h intervals, 12 h following exosome infusion. A total of four antibody injections were given. In control studies in vitro (not shown), we found that 20 *μ*g of each serum attenuated by >80% the ELISA signal from 5 *μ*g recombinant cytokine.

### In vitro tumor cell migration assays

Where migration/invasion of EMT6 or 4THM cells was assessed in vitro, Boyden chambers (EMD Millipore Corporation, Etobicoke, Canada) with pore size 8 *μ*m were used. Single cell suspensions of tumor cells (2 × 10^5^) were incubated in serum‐free medium in the upper chamber, with the lower chamber including complete medium with fetal calf serum (and 5% mouse serum [or exosomes], cytokines, and/or anticytokine antibodies [10 *μ*g/mL] as noted in individual studies). After 18 h, migrating cells were detached from the underside of the semipermeable membrane by incubation in cell detachment buffer, centrifuged, and counted manually.

### Tumor explant culture and multianalyte ELISArray

Individual tumors were removed at ~1 cm^3^ size (~14 days post injection), washed three times with cold PBS containing 100 IU penicillin and 100 *μ*g/mL streptomycin, cut into small fragments (<1 mm^3^) using scalpel blades, and cultured in 500 *μ*L of supplemented RPMI 1640 culture medium at 37°C with 5% CO_2_ humidified air for 24 h. Thereafter supernatants were collected and particulate material removed by centrifugation for 10 min at 2500*g*. The supernatants were used for cytokine and chemokine analysis using a multianalyte ELISArray kit (Qiagen, Mississauga, ON). Twelve proinflammatory cytokines and chemokines were examined in the supernatants, including IL‐1*β*, IL‐4, IL‐6, IL‐10, IL‐12, IL17A, IFN‐*γ*, TNF*α*, TGF*β*, MCP1, MIP‐1*α*, and MIP‐1*β*. Capture antibodies for the 12 cytokines and chemokines were coated on one ELISArray microplate and 50 *μ*L of samples were added to the wells of the plate. After 2 h incubation and exhaustive washing to remove unbound proteins, 100 *μ*L of biotinylated detection antibodies were added. Thereafter, an avidin–horseradish peroxidase conjugate was added after 1 h incubation. After further washing substrate solution was added. A stop solution was added after 30 min and the absorbance at 450 nm was read. In some studies conventional commercial ELISA kits (BioLegend, Dedham, MA, USA) were used to quantitate absolute cytokine levels (see Fig. 5).

## Results

### Comparison of CTCs in EMT6 and 4THM WT tumor‐bearing mice

Unlike 4THM 15, EMT6 tumors growing in wild‐type (WT) mice had elevated expression of cell surface CD200, and this elevated expression of CD200 increased local and metastatic growth of EMT6 13, 15. Metastasis of EMT6 was decreased in CD200R1KO while 4THM grew more rapidly in CD200R1KO mice, with increased visceral metastases, despite there being no obvious difference in tumor infiltrating cell populations 15. We asked whether other surrogate markers for metastatic tumor growth might help explain the difference in metastases of these two tumors in WT and CD200R1KO. CTCs, cells which have “sloughed off” from the primary tumor and are released into the peripheral circulation, are thought to represent a source of most metastases 21. While their absence of CD45 expression is an important negative criterion, positive selection of CTCs is more controversial 22, and accordingly we used CD45^−^ cells isolated from PBL of both EMT6 and 4THM tumor bearers, and measured the frequency of CTCs in WT/CD200R1KO by limiting dilution as described in the Materials and Methods section.

**Figure 1:**
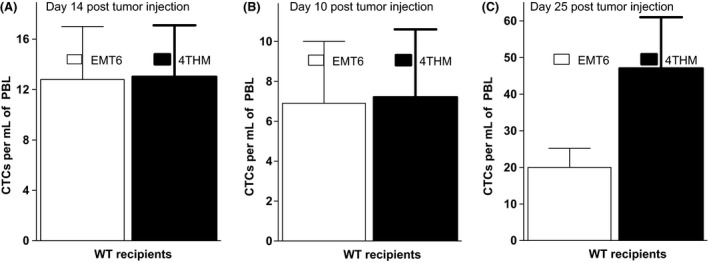
Frequency of circulating tumor cells (CD45^−^) per mL of peripheral blood in mice at 10 (B), 14 (A), and 25 days (C), post 2 × 10^5^ tumor cell injection. All groups contained five mice. CD45^−^ cells were obtained from individual mice by depletion on CD45^+^ magnetic bead columns (Miltenyi), and cloned in 96‐well microtitre plates on feeder layers with 10^5^/well irradiated (3000 Rads) homologous tumor cells. Tumor cell clones were scored in individual wells at 21 days post culture. Data show mean ± SD for each group.

Using day 14 EMT6 or 4THM WT tumor‐bearing mice, we found that the frequency of CTCs in both tumor models was essentially the same (~10–15 CTCs/mL PBL; Fig. 1B). This was somewhat surprising, since analysis of DLN metastases in the same animals had shown a 5‐ to 10‐fold greater frequency in 4THM tumor bearers 23. Analysis of CTCs in PBL at earlier times (d10; Fig. 1A) showed again no differences in the two models (~5–10 CTCs/mL PBL), although at later times (d25; Fig. 1C) substantially more CTCs/mL PBL were observed in 4THM (35–65) than in EMT6 20, 21, 22, 23, 24, 25 tumor bearers, consistent with the far greater tumor burden (local and lung/liver metastases) in the former mice.

### CTCs and microscopic DLN metastases for EMT6 and 4THM in WT/CD200R1KO and CD200^tg^

**Figure 2:**
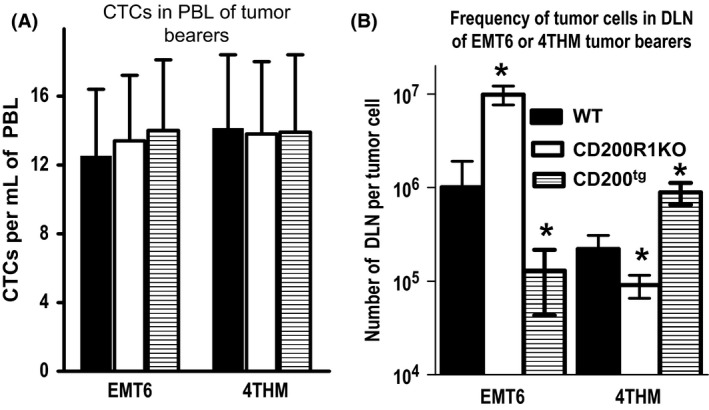
Frequency of circulating tumor cells (CTCs) in peripheral blood (A) (see Fig. 1) or in draining lymph node (DLN) (B) at 14 days post tumor inoculation of EMT6 or 4THM tumor cells into 5/group wild‐type (WT), CD200^tg^ or CD200R1KO mice. Data show mean + SD for each group. *P < 0.05 relative to WT control.

A dichotomy between the measured frequency of CTCs in PBL and of cloneable tumor cell micrometastases in DLN was seen for these two tumors when comparison was made between WT versus CD200R1KO or CD200^tg^ mice (Fig. 2). As reported earlier 14, there was a marked decrease of EMT6 tumor cells detected in DLN of CD200R1KO mice, with corresponding increases in 4THM mice. Thus, >10‐fold more DLN cells were cultured for each cloneable metastatic EMT6 tumor cell in CD200R1KO versus WT mice (left hand groups, Fig. 2B: 10^7^ vs. 10^6^ DLN cells, respectively). In contrast an increased frequency of EMT6 metastatic cells was seen in DLN of CD200^tg^ mice (now one tumor cell detected for ~2 × 10^5^ DLN cells—see left hand side of Fig. 2B; 13). Essentially the opposite was the case with 4THM tumors, with increased frequency of metastasis in CD200R1KO and a decrease (relative to WT) in CD200^tg^ mice (see right hand side of Fig. 2B). The relative changes in DLN metastases in the three mouse strains correlated well with macrometastases measured in liver/lung 23. However, there was no significant change in CTCs in any of the mice used, regardless of tumor injected (Fig. 2A). We concluded that the effect of CD200:CD200R interactions on differences in metastases seen in these two tumor models could not be explained by a difference in seeding into the circulation, and might instead reflect regulation of the environment (in the DLN) leading to an altered frequency of tumors cloned from that location.

### A role for serum‐derived factors/exosomes in altered metastases seen in WT/CD200R1KO mice

**Figure 3:**
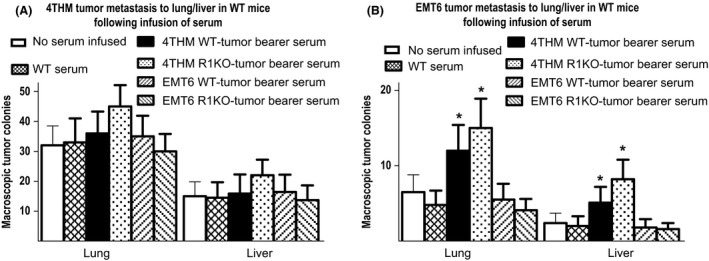
Macroscopic tumor colonies in lung/liver of wild‐type (WT) mice receiving tumor cells and six injections of 150 *μ*L serum i.v. from the sources shown at 48 h intervals, beginning on the day of tumor injection. Primary tumors were resected at 14 days and tumor colonies evaluated 10 days later in all mice. Data show mean ± SD for each group. *P < 0.05 compared with control receiving no serum infusion.

**Figure 4:**
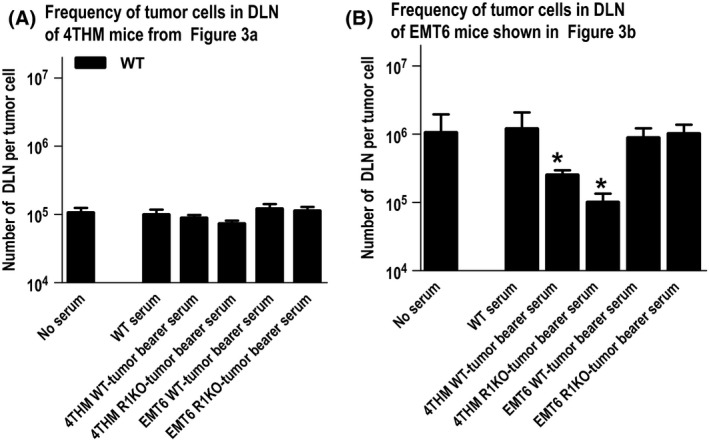
Frequency of tumor cells in draining lymph node (DLN) of mice shown in Figures 3A and B. Data show mean ± SD for 5 mice/group. *P < 0.05 compared with controls with no serum injections.

We harvested serum from day 14 4THM or EMT6 tumor‐bearing WT/CD200R1KO mice, and infused 150 *μ*L of serum i.v. into naïve WT BALB/c mice six times at 48 h intervals. On the day of initial infusion mice received mammary fat pad injection of 4THM or EMT6 tumor cells. Control groups in all cases received either no serum infusions, or serum from control WT (tumor free) mice. Primary tumors were resected from all mice at day 14, and mice were sacrificed 10 days later to enumerate macroscopic lung/liver metastases (Fig. 3A and B). In addition, microscopic DLN metastases in the same animals were determined as in previous figures, by limiting dilution (Fig. 4A and B). Infusion of serum from 4THM tumor‐bearing WT or CD200R1KO mice increased both macroscopic (to lung/liver; Fig. 3B) and microscopic metastasis (to DLN; Fig. 4B) of EMT6 tumor cells—thus in DLN, fewer DLN cells were cultured to reveal each tumor colony. In contrast, these same sera exerted no significant effect on the frequency of metastasis of 4THM tumors (Figs. 3A and 4A). These data suggest the presence of a factor(s) in sera of WT/CD200R1KO 4THM tumor bearers which can increase the metastatic potential of poorly metastatic EMT6 tumors. In addition, since the frequency of 4THM tumor metastases was unaffected by infusion of plasma from the poorly metastatic EMT6 tumor (Figs. 3A and 4A), our data do not imply the presence of a “metastatic protection” factor in such sera.

**Figure 5:**
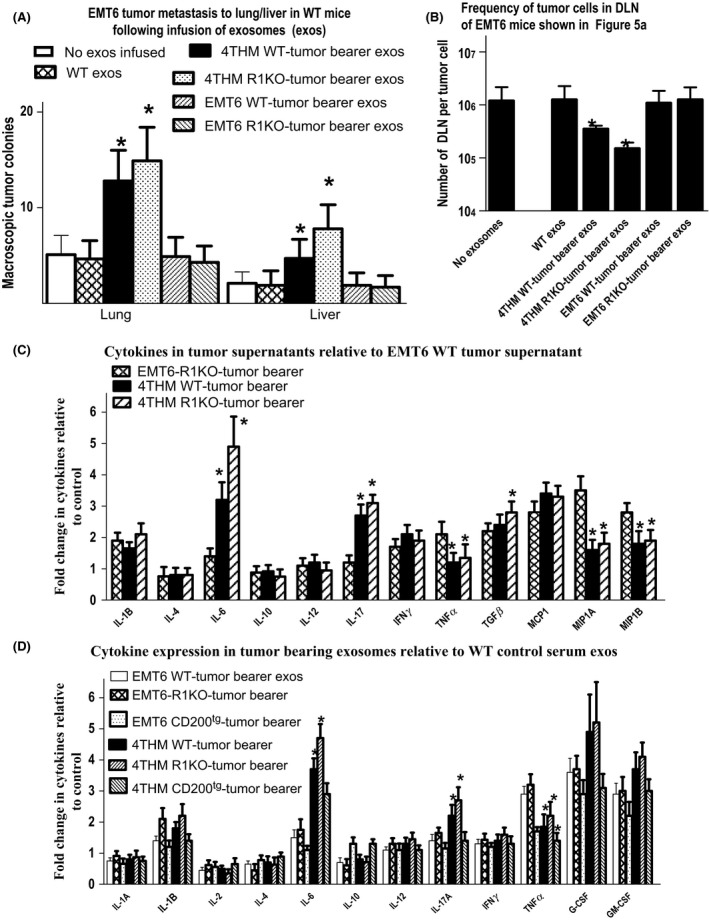
Frequency of macroscopic tumor metastases (liver/lung) (A), or of microscopic tumor metastases (assayed by limiting dilution in draining lymph node [DLN]) (B) in mice initially receiving EMT6 tumor cells and serum‐derived exosomes (exos) from wild‐type (WT) or CD200R1KO tumor‐bearing mice. As in Figure 3, exosome infusions were given at 48 h intervals, and all tumors were resected at day 14, with mice sacrificed for analysis of metastasis 10 days later. Data are pooled from two studies (each with 4 mice/group). In (C) and (D) data show means ± SD for commercial ELISA arrays detecting cytokines/chemokines in supernatants of tumor cells harvested from wild‐type (WT) or CD200R1KO mice (C), or in exosomes harvested from serum of the tumor bearers (D). Each bar in each of panels C and D represents a mean of five independent measurements. *P < 0.05 compared with control groups receiving WT exosomes (A and B), or compared with EMT6 WT tumor explant culture (C), or compared with exosomes from control nontumor‐bearing mice (D). The corresponding maximum cytokine levels of IL‐6, IL‐17, and TNF*α* measured in tumor explant or exosome samples were 550 ± 65, 375 ± 40, 415 ± 65 pg/mL and 510 ± 55, 295 ± 45, 365 ± 55 pg/mL, respectively. All cytokines were measured using conventional ELISA kits (BioLegend, USA).

In a follow‐up study we asked whether exosomes isolated from serum of 4THM or EMT6 tumor‐bearing mice, compared with nontumor‐bearing exosomes, could substitute for whole serum in adoptive transfer of increased metastasis of EMT6 tumors in WT mice. Data shown in Figure 5A and B confirm that exosomes isolated from both WT and CD200R1KO 4THM tumor bearers could transfer increased macro‐ and micrometastatic potential for EMT6 in WT mice when used at equivalent concentrations to the serum from which they were derived.

Given evidence elsewhere that cytokines/chemokines might be particularly implicated in metastatic growth 15, we also measured, using commercial ELISA cytokine arrays, the cytokine profiles for both tumor explants (Fig. 5C) and exosomes (Fig. 5D) in these models. Our data show an increased IL‐6 and other inflammatory cytokines from both exosomes and tumors of 4THM tumor bearers with the most elevated levels seen in CD200R1KO and the lowest in CD200^tg^ mice, data consistent with our earlier reports 15. We next explored whether these cytokine changes might be in part at least responsible for the altered metastasis of EMT6 tumor cells seen following serum/exosome infusion.

### Neutralization of IL‐6 and IL‐17, but not TNFα, attenuates increased metastasis of EMT6

**Figure 6:**
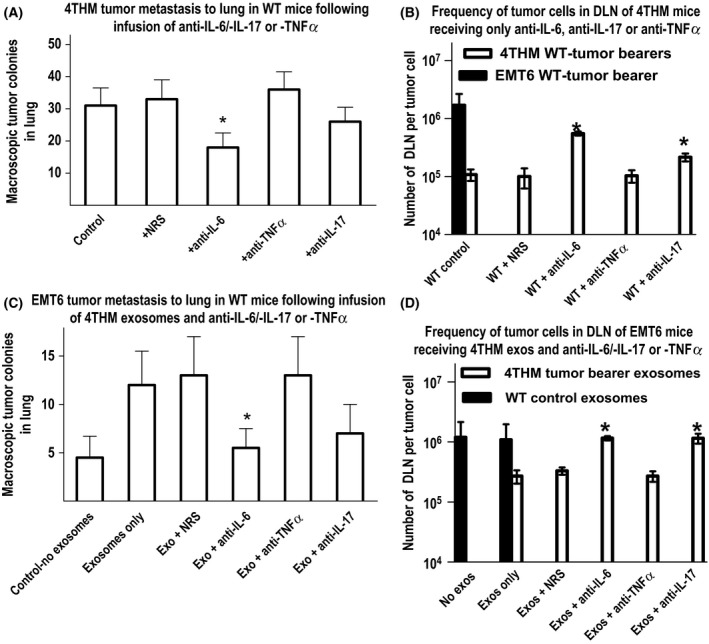
Attenuation of increased frequency of macroscopic (to lung) (A) and microscopic tumor metastases (assayed by limiting dilution in draining lymph node [DLN]) (B) by anti‐IL‐6/anti‐IL‐17, but not anti‐TNF *α*, in wild‐type (WT) mice receiving 4THM tumors (no additional exosomes). The DLN metastatic frequency in mice receiving EMT6 tumor cells is shown as a filled‐in histogram to the far left in panel B. (C, D) Similar data for mice initially receiving EMT6 tumor cells and serum‐derived exosomes (exos) from 4THM CD200R1KO tumor‐bearing mice. As in Figure 3, exosome infusions were given at 48 h intervals, with four 50 *μ*g/mouse doses of antibodies (or NRS or anti‐TNF *α* as controls) given at 60 h intervals. In all cases, tumors were resected at day 14, and mice sacrificed for analysis of metastasis 10 days later. Data are pooled from two studies (each with 4 mice/group). *P < 0.05 compared with NRS controls.

Evidence elsewhere suggests a role for inflammation and inflammatory cytokines on both tumor growth and metastasis 24, 25. IL‐6/IL‐17 production is increased in 4THM tumors themselves, and in exosomes from tumor‐bearing mice (Fig. 5). To investigate whether this might mediate the enhanced metastatic capacity of 4THM, and the transfer of increased metastatic capability to EMT6 tumors (Fig. 5), we used neutralizing antibodies to those cytokines to attenuate metastasis of 4THM and the effects of exosomes from 4THM mice on enhanced metastasis of EMT6. In Figure 6A, a series of WT mice received 4THM tumors only (no exosomes) with/without four infusions of 50 *μ*g/mouse rabbit anti‐mouse IL‐6, anti‐IL‐17, or anti‐TNF*α* antibody or normal rabbit serum as control. In Figure 6B, EMT6‐injected WT mice received exosomes from 4THM CD200R1KO tumor‐bearing mice along with antibody injections. Tumors were again resected at day 14, and mice sacrificed 10 days later. At this time lung tumor colonies were evaluated macroscopically, and DLN metastases assayed by limiting dilution culture. Anti‐IL‐6, and to a lesser degree anti‐IL‐17, led to attenuation of both lung and DLN micrometastases in 4THM tumor bearers. In addition, both anti‐IL‐6 and anti‐IL‐17 produced significant attenuation of the increased EMT6 DLN metastasis seen following infusion of 4THM exosomes into mice, while anti‐TNF*α* produced no measurable effect.

**Figure 7:**
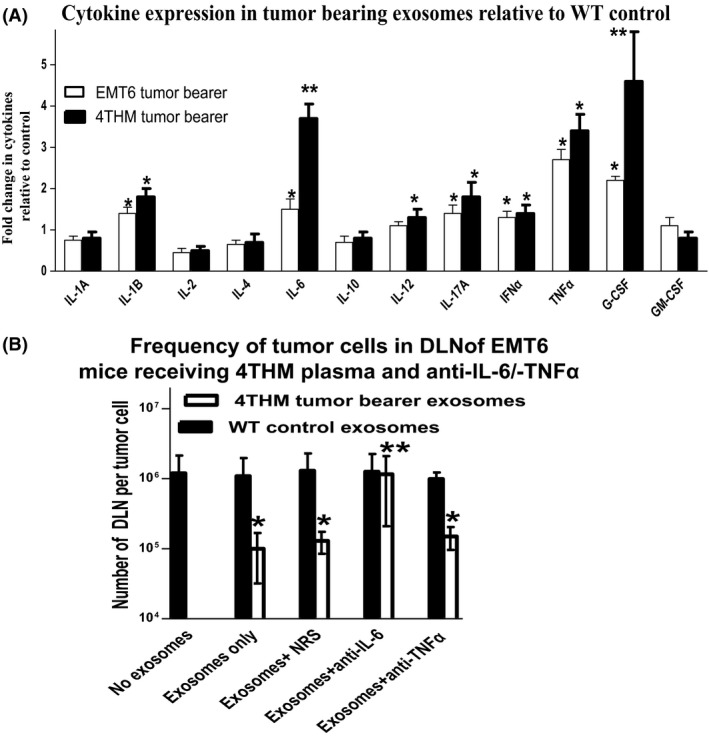
Effect of serum or exosomes (equivalent to 5% serum concentration in vitro) from wild‐type (WT) or 4THM mice, or recombinant cytokines (IL‐6/IL‐17: 5 ng/mL) on migration of 4THM (A) or EMT6 (B) tumor cells in Boyden chambers over 18 h. Cells (2 × 10^5^) were included in the upper chamber only (an 8‐*μ*m pore size filter was used). At 18 h, cells were detached from the lower surface of the filter, concentrated by centrifugation, and counted. Data show mean ± SD for triplicate samples. Data along the abscissa show the culture conditions used in the lower chamber. In some cultures, anti‐IL‐6/anti‐IL‐17 antibodies (10 *μ*g/mL) were also included in the lower chamber. *P < 0.05 compared with cells migrating in medium only; ^*δ*^ *P* < 0.05 compared with equivalent cultures without anti‐IL‐6.

In a final series of studies we asked whether tumor‐derived serum/exosomes, or recombinant cytokines alone, could alter migration of 4THM or EMT6 tumor cells in vitro. In addition, we asked whether any altered migration was in turn modified by inclusion of anticytokine antibodies in the in vitro (Boyden) migration chambers. Data shown in Figures 7A and B (for 4THM and EMT6, respectively) showed a modestly increased migration only of EMT6 tumor cells in the presence of 4THM tumor‐bearer serum/exosomes and recombinant IL‐6 (not IL‐17), with attenuation of migration of both 4THM and EMT6 by anti‐IL‐6 in vitro.

## Discussion

Improved understanding of cancer immunotherapy must take account of the fact that most strategies will be directed to patients with pre‐existing tumors who may already express some degree of tumor antigen tolerance. A number of protocols have been developed with this in mind, including those directed at manipulating dendritic cell development to improve induction of cells at inducing T‐cell immunity rather than tolerance 26, as well as targeting how T cells traffic to the tumor site, or how their early exhaustion and/or immunosuppression within the tumor microenvironment might limit immune T‐cell efficacy. Attenuation of tumor cell‐mediated immunosuppression can occur through release of soluble factors (e.g., TGF*β*, IL‐6, IL‐10, VEGF, etc.) which directly or indirectly affect T‐cell immunity (e.g., through augmented induction of myeloid suppressive cells, MDSCs which block production of mature DCs), and/or through tumor‐cell‐mediated enhancement of immunoregulatory pathways 7, 20. Currently attention has focused on understanding the mechanism(s) of action of ligand:receptor pairs which regulate immune reactivity at key “checkpoints.” Within the tumor environment overexpression of molecules expressed by T cells including cytotoxic T‐lymphocyte antigen 4 (CTLA‐4), programmed death‐1 (PD‐1), lymphocyte activation gene‐3 (LAG‐3), and T‐cell immunoglobulin and mucin‐containing protein 3 (Tim‐3) have been linked to hyporesponsiveness in T‐cell activity (T‐cell exhaustion). mAbs targeting either the ligands or their receptor(s) have been studied for their clinical efficacy with some notable recent positive results 8, 9.

Our own interest in this area was fueled by evidence from the transplant field that engagement of the CD200:CD200R axis caused profound anti‐inflammatory and immunosuppressive effects 27. We hypothesized that such an interaction, if operative during tumor growth, would result in attenuated host resistance and death 10. Complications to this simple interpretation of our data came with evidence that the growth and metastasis of 4THM and EMT6 tumors in mice showed different responses to alterations in the inflammatory environment regulated by CD200:CD200R, with attenuation of CD200:CD200R signaling as predicted decreasing metastasis of EMT6 in CD200KO or CD200R1KO mice 14, 23, while metastasis of 4THM was actually increased in CD200R1KO 15. Importantly, when we measured the frequency of CTCs in WT, CD200^tg^ or CD200R1KO tumor bearers with EMT6 or 4THM tumors, we observed no obvious difference between the two tumors, or within the various mice (Fig. 2A), despite clear difference in the micrometastases (assessed by limiting dilution analysis of DLNs; Fig. 2B). Differences in frequency of tumor cells in DLN were well correlated with subsequent macrometastases measureable in lung/liver 23, leading us to conclude that the difference in metastases seen in these two tumor models in mice with altered CD200:CD200R signaling did not reflect altered seeding into the circulation, but rather a differential conditioning of the microenvironment making it permissive for development of metastases.

Interestingly, serum from 4THM tumor‐bearing WT/CD200R1KO mice facilitated increased EMT6 metastasis in WT mice (Figs. 3B and 4B), with CD200R1KO‐derived serum being slightly superior, although the converse effect (decreased 4THM metastasis using infusion of EMT6 tumor bearer serum) was not observed (Figs. 3A and 4A). Similar effects were produced following infusion of serum‐derived exosomes (particularly CD200R1KO‐derived exosomes) from the same mice (Fig. 5A and B). Differences were also seen in the cytokine production profile from both tumor cells themselves (Fig. 5C) or in serum exosomes of tumor‐bearing mice (Fig. 5D), with enhanced IL‐6 and IL‐17, and moderately decreased TNF*α* in 4THM versus EMT6 tumor bearers. These differences were most evident in samples obtained from CD200R1KO tumor bearers. As noted before, epithelial‐to‐mesenchymal transition is regulated by an inflammatory cytokine milieu 16, 17, 18, 19, with many cells within the tumor microenvironment, including tumor‐associated macrophages (TAMs), dendritic cells, and tumor infiltrating lymphocytes as potential sources of these inflammatory cytokines. High IL‐6 levels and activation of IL‐6:STAT3 signaling has been reported to induce a cancer stem cell phenotype to nonstem cells 28, while IL‐6 trans‐signaling (through IL‐6:IL‐6R) has itself been implicated in 4T1 metastasis 20. Importantly, a recent study has also suggested a crucial role for increased IL‐17 levels in murine breast cancer metastasis to bone/lung, perhaps through altered IL‐6 and/or chemokine expression 29.

In a final series of studies to clarify the potential role of the cytokine changes observed in the tumor models, and specifically the role they may play in tumor metastasis, we studied the effect of Anti‐IL‐6, anti‐IL‐17, and anti‐TNF*α* on metastasis of 4THM tumors (Fig. 6A), and on the 4THM exosome induced enhanced metastasis of EMT6 tumors in WT mice (Fig. 6B). Further studies in vitro (Fig. 7) explored the ability of 4THM serum/exosomes or recombinant cytokines to alter cell migration in a Boyden chamber, and the ability of anticytokine antibodies to attenuate such effects. These data show clearly that both anti‐IL‐6 and anti‐IL‐17 diminish 4THM metastases in vivo, and abolish the adoptive transfer of increased EMT6 metastasis in WT mice by 4THM CD200R1KO exosomes. As anticipated, 4THM cells migrated in a superior fashion to EMT6 in a Boyden chamber (compare medium control to far left in each of panels Fig. 7A and B). However, only anti‐IL‐6 produced a significant attenuation of migration of tumor cells in vitro, and enhanced tumor cells migration was seen only using EMT6 tumor cells incubated with 4THM serum, exosomes, or recombinant IL‐6. Preliminary studies (R. M. G., unpubl. data) suggest that recombinant IL‐6 infused daily in vivo to WT mice also increases EMT6 metastasis to DLN as assayed by limiting dilution analysis.

It is worth noting that earlier studies in the EMT6 model had reported that anti‐TGF*β* antibodies could increase metastasis to DLN 13. TGF*β* is produced by multiple cells types within the tumor microenvironment, including, but not limited to, macrophages, endothelial cells, and fibroblasts, and its release has been reported to favor EMT in cancer cells 19, with localized TGF*β* expression providing a link between EMT and CSC induction 30. Such effects would predict that anti‐TGF*β* might attenuate, not increase, metastasis in breast cancer, and indeed, in 4THM mice, this seems to be the case (Gorczynski, unpubl. data). The anomalous results seen in the EMT6 model highlight the complexity of understanding outcomes in tumors where host protective responses are mitigated by suppression of inflammation/immunity (as we presume occurs in the EMT6 model), versus scenarios where host inflammatory responses, perhaps through increased EMT/CSC induction, increase tumor growth (in 4THM). Thus, the principle effect of anti‐TGF*β* in EMT6 tumor‐bearing mice may lie at the host (not tumor) level to attenuate host protection and thus enhance metastasis indeed the enhanced metastasis following anti‐TGF*β* in EMT6 mice 13 was associated with increased MDSCs in the tumor, a population relevant in promoting EMT6 20, 31. We hypothesize that in 4THM tumor‐bearing mice in contrast, the primary effect of TGF*β* is a direct one on tumor cells, increasing EMT and CSC induction to promote metastasis.

We have recently shown that by combining conventional surgical therapy with immunotherapy targeted at CD200:CD200R immunoregulation, long‐term cure of EMT6 tumors in mice is possible 32. While EMT6 tumor‐bearing mice can also be effectively treated with combination chemotherapy (paclitaxel and anti‐VEGF), these mice do not display sterile immunity and are not protected from rechallenge with the same tumor, implying an ongoing susceptibility to metastatic recurrence 23. A phase I clinical trial reported a positive response to anti‐CD200 therapy in a mixed population of lymphoma/leukemia cancer patients 33. A number of trials have begun to explore the effect of blockade of IL‐6/STAT3 signaling in solid tumors 34, 35. It remains to be seen whether these treatments add significantly to the current armamentarium, and if so, the mechanism(s) by which they might produce such effects (e.g., through altered host resistance and or EMT/CSC development).

## Conflict of Interest

None declared.
